# Parental Corrections

**Published:** 1864-03

**Authors:** 


### HALL'S JOURNAL OF HEALTH.

Our Legitimate Scope is almost boundless: for whatever begets pleasurable and harmless feelings, promotes Health; and whatever induces disagreeable sensations, engenders Disease.

WE AIM TO SHOW HOW DISEASE MAY BE AVOIDED, AND THAT IT IS BEST, WHEN SICKNESS COMES, TO TAKE NO MEDICINE WITHOUT CONSULTING A PHYSICIAN.

Vol. XI.] MARCH, 1864. [No. 8. 3

### PARENTAL CORRECTIONS.

HISTORY records that one of the pyramids was built at the cost of a kiss of the king's daughter, for every man who furnished a stone for its construction. Walking down-town the other day, with a retired merchant of great social and private worth, the remark was made as we passed a splendid hotel, its white marble front glistening in the morning sun, "Every stone in that building cost the ruin of a young girl, if newspaper report be true;" as the builder owned a large property in Mercer and Church streets, the locality of assignation-houses.

"What, Doctor, do you think is the chief source of supply for the victims of the great social evil of large cities?"

"Unhappy homes," was the instinctive reply.

A distinguished judge once said, at the close of a long life, that most of all the male criminals brought before him were found on investigation to have made the first steps toward ruin between the ages of eight and sixteen.

Putting all these things together, the inference may be safely drawn, that a large share of all the unhappiness and crime in the world arises from the character of parental management, its failure to be of a kind to make home the happiest place for the child. If children are indulged too much, they soon begin to feel the least restraint, the slightest opposition to their wishes, an intolerable burden, and their spirits chafe like a caged tiger. Too much restraint, on the other hand, an incessant fault-finding, an everlasting laying down of rules and regulations, intemperate chidings, altogether disproportioned to the offense; a habitual rehearsal of the faults of children to all visitors indiscriminately, and ruthless reprovals in the presence of others, their friends and playmates — each and all of these barbarities, as they may be very properly termed, have the very natural effect to sour the young heart, to make it feel as if the parent, who ought to be the best friend, is really the greatest tormentor; then a feeling of defiance and desperation succeeds, and by degrees the settled purpose is formed, of seeking means to escape from a control which has now grown up to be considered arbitrary and tyrannical to a degree not to be borne another hour; and often, in a fit of passion, a step is taken which can never be recalled. When a daughter begins to feel, with or without cause, that she has not her mother's sympathies, that her mother does not enter into her years, and is deficient in that tenderness which is naturally looked for, she turns all the more eagerly to the attentions, the deference, and the consideration which the young man shows her, and before she is aware of it, she is ruined forever!

The undutiful step-mother has driven countless thousands from once happy and virtuous homes to crime and infamy Harsh, unfeeling, inconsiderate teachers have many times driven the young to desperation or hopelessness. In the last year's Journal three articles, with most impressive illustrations, were published, and now three more are given, in the hope of compelling a very general attention to this most important subject. The first incident occurred within a few miles of our birthplace.

"Some three years ago a household in the city of Covington was thrown into commotion by the sudden disappearance of a daughter twelve years of age. She was tracked to the ferry-boat, but whether she passed safely over or had been drowned was not discovered. Patient and anxious waiting brought no tidings of her. The frenzied and unhappy father, although in moderate circumstances, sought the newspaper-offices, and advertised a reward of one thousand dollars to whoever should restore his missing child. All proved unavailing. Some time afterward the corpse of a young lady was found in the river near Vevay, Indiana, and hearing of it he went there, but it was not his daughter.

"Time went on, and no tidings came of the lost child. She was dead to them, but they could not visit her grave. About twelve months since, the stricken family removed to Mexico and took up their abode in a country foreign in language and customs, in features and in habits, from that in which they had met with their great loss. It might wear away their thoughts from sadly ruminating on the past, and enable them, in a region more devoted to religious duties, to look more hopefully toward the great future. There they still are.

"About a week since a steamer arriving from Memphis was crowded with passengers, who were upon the guards straining their eyes to gather into one look the multitudinous objects which throng the public landing. One, however, a young girl budding into womanhood, sought the outer rail and looked wistfully over the naked shore of Covington to where, hid away under a clump of trees, was the cottage of her childhood, hoping in vain to see the curling smoke announce to her a warm welcome within. Quickly she passed over the ferry where long since she had disappeared. No one noted or knew her, and she went without interruption to the door of her father's house. It answered not her knock; weeds had grown up rank and rough where she had left flowers, and no signs of human life were to be found there.

"It was the turn now of the wayward child to weep, and when by inquiry she found how far and almost hopelessly she was separated from her parents, she began to feel desolate. Piqued at some chiding or some punishment of her mother, she had gone upon a steamboat, where a female passenger hired her as a nurse. After a little while the war broke out, stopping all intercourse with the South by the river, and, though she soon found that untried friends but seldom prove steadfast in trouble, and that the harshness of a parent is melting kindness besides that of a stranger, yet she was unable until lately to return. A kind lady of Covington has given shelter to the wanderer until her return is made known to her parents."

### THE HARSH TEACHER.

Says an exchange: "We listened, the other day, to an eminent divine, one of America's most gifted and honored sons, as he gave some account of the 'wrongs of his boyhood.'

"'I went away to school,' said he, 'when I was seven years old. My teachers never understood me; my first teacher assumed that nothing was easier than to understand children. Hence he never took pains to study the character of a child.'

"'You have blotted your book, sir—how is that? Do you mean to disobey me? Have I not told you that I would have clean writing-books?' said my master.

"'I have *not* blotted my book,' said I stoutly.

"'Who has blotted it, then? No one has had it but yourself. Do you accuse any one else?'

"'I do not accuse any one, and I have not blotted my book.'

"I spoke in good faith, though impudently. I had no knowledge of having blotted my book.

"'Hold out your hand, and be punished for disobedience and lying.'

"I held out the hand that my mother had so softly kissed. I was not eight years old. The master ferruled me till he was tired, and I never shed a tear. My eyeballs seemed on fire. The teacher rested, and then whipped me again; I did not weep or cry out.

"'You shall beg to be let off, sir,' said he. I did not beg. I endured all he chose to inflict, and he was obliged to leave me at last, worn out by my obstinacy.

"'The worst boy I ever saw,' said he. 'You will come to the gallows yet. You have not human feelings.'

"I looked at my swollen and discolored hand. Oh! if any one had kissed that little hand instead of beating it, I could have hid my face in his bosom, and wept for every insult I had committed or ever should commit. I believe I registered a vow in heaven, then, to be always kind to little children."

### AN OLD MAN'S STORY.

"I am an old man; yet it seems a very short time since I climbed the tall poplar-tree that grew before the vicarage, in search of the starling's nest. I can fancy I hear the shout that greeted my descent with the long-coveted prize, and feel again the crimson mounting to my cheeks as it did when, turning to the vicarage, I saw an expression of pain on the pale face of my father as he stood at the study-window.

"It seems to me but yesterday since I stood in the center of that group of lads, and now

'They are all gone, the old familiar faces.'

"Dick, the surgeon's son, died many years ago in India. Harvey Vernon, the bravest of them all, was slain on the field of Waterloo; and when the village bells rang for the victory, the rudest fellow in the village was touched as he passed the Grange and saw the blinds down and knew of the breaking heart of old Widow Vernon.

"It was a sad day for us at the vicarage, especially for Emily. My father staid in his library all day, though I do not think he read a page in any of his books — even in his favorites, Sophocles and Horace.

"Emily and my mother were in my mother's chamber all the day. From that day Emily gradually drooped and faded. Her beautiful face grew more exquisitely beautiful — her dark deep eyes became more full and lustrous, but they wandered restlessly, as though seeking some missing resting-place; her golden hair (I have still a thick lock of it amongst an old man's memorials of other days, 'the days of auld lang syne') hung more carelessly about her shoulders, and her pale cheeks were suffused with a rosy tint that gradually deepened into a burning crimson, while her sweet voice sunk almost into a whisper. As I looked at her, her startling beauty reminded me of the language of the book my mother used to read to her as she lay on the couch in the drawing-room. Her 'face was as the face of an angel.'

"Ah me! how I am wandering from the circumstance I sat down to write about; but you must forgive an old man, for whenever I think of Emily, it is always so. Let me see—yes, I remember perfectly.

"It was Christmas eve, in the year 1791, and the snow had been falling heavily all the day, blotting out the hedges and walls which surrounded the vicarage, and burying the sun-dial that Willie and I had carved with great pains during the long winter evenings.

"I had come from my father's study, where I and Willie had been having our usual lesson in Latin. Willie was a high-spirited lad, of a very loving and affectionate disposition; though, when excited or in a passion, his temper was fearful to behold, and his eyes flashed with a strange light that made us all tremble, except my father.

"It was some time before my father came down; but when he did, we heard him lock the study-door after him, and he came down alone. He looked very stern and angry; he was in one of those moods which sometimes took possession of him when he was disturbed. Though my father was always silent when in these moods, yet I always thought there was a vivid resemblance between them and Willie's outbreaks of passion.

"'Willie will not come down to-night,' said he; 'I have left him in the study, with a lesson that will keep him all night.'

I thought I saw a tear start from my mother's eye, as she turned her face to the window and looked out upon the snow, which still continued to fall heavily.

"It was the anniversary of Emily's birthday, and we were expecting a party of her young friends, (children of the neighboring gentry,) to pass the evening at the vicarage.

"It began to grow dark about four o'clock, and then our company began to arrive. There were, first, the children of 'Squire Harcourt, who came wrapped in soft furs and shawls, in the old-fashioned cozy family carriage, with its couple of docile grays. Then came Harry Vernon, and his sisters, Emily and Agnes; and, as the time wore on, about a score of young people were assembled at the vicarage. It was a merry party. My father, whom it would be an injustice to represent as an unkind man, threw himself into the spirit of our merriment as though he had been one of us. The furniture, excepting the old-fashioned piano, had been removed from the drawing-room, and it and the sitting-room had, by the removal of a partition, been thrown into one, making a large and commodious room, which had been plentifully hung with holly and other evergreens. The red berries gleamed like tiny masses of fire beneath the dark green glossy leaves, and here and there my sister's hands had gracefully arranged bunches of many-colored ribbons.

"Many inquiries were made for Willie, and for a moment or two a shadow seemed cast upon the pleasure of the children when they were told that Willie, the presiding spirit of fun in every juvenile party, would not be with them; but all feeling of disappointment vanished as the time wore on—except from one gentle, loving spirit.

"I knew that my mother was thinking of the dear boy in the room above us, for Willie was my mother's favorite. She was thinking of a handsome face pressed against the door, and of a tiny ear close to the key-hole, listening to the voices of the merry groups below. She knew these sounds would be exquisite torture to the prisoner. She knew how that quick, eager spirit would fret in the study above, like a wild bird in a cage.

"Sometimes I saw her whisper to my father, and then his face grew hard and dark, and my mother's yet more sad and pained.

"My sister played, with exceeding grace, some simple airs upon the old piano; and then, the boys choosing their partners from the little maidens who stood with eager, blushing faces and beseeching eyes, beneath the holly in a corner of the room, the dance began.

"While this was going on I saw my father put something into my mother's hand; it was the study-key. With a grateful smile—oh! how sweet that smile was!—she left the room. I stole after her to the foot of the wide, old-fashioned staircase; I saw her glide swiftly up the stairs; and I could hear when she unlocked the door; and when she opened the door to pass in, the moonlight streamed brightly through the doorway on to the dark landing, and as its light fell on the face of the old clock which stood there, I saw it wanted but a few minutes of ten o'clock.

"I had not stood more than a minute at the foot of the stairs, when I heard my mother cry: 'Willie!' Then I heard a piercing scream, and she suddenly passed me, her face white as the snow that lay outside on the steps, and rushing into the room where my father was playing with the children, went straight up to him, and crying, 'Willie's gone! O Willie, Willie darling!' fell fainting at his feet.

"My sister immediately left the piano, and with the aid of some cold water my mother was restored very soon. Of course, this put an end to the festivities, and the children were soon on their way home, except Harry Vernon, who staid to assist in the search for the missing boy. Afterward my mother told us, that as she was endeavoring to amuse a group of the younger children, she heard Willie's voice distinctly calling, 'Mamma! mamma!' She instantly got the key, as I have before related, and went up to the study. As soon as she opened the door, she felt the window was open, by the rushing of the cold frosty air past her. The instant she entered the room she felt a tremor seize her! Why did not Willie spring to meet her? She felt in a moment that Willie was not there! The study-lamp was flickering out; there stood my father's easy-chair opposite a table on which lay his books and manuscripts, and amongst them poor Willie's soiled and hated Latin Grammar.

"He must have climbed down the side of the old house, by the aid of the ivy-stems which grew up to the pinnacles of the gables on to the top of the antique portico, and from thence have leaped to the ground. Willie, agile as a squirrel, could easily have accomplished this.

"In a few moments from the discovery of his absence, we—that is my mother and father, Harry and myself, and two servants, one of them old Walter, who passionately loved Willie—were out in search of the missing one.

"The snow was still falling heavily, but by the light of the moon, which was at full, we could see almost as distinctly as by daylight.

"Strange to say, my mother went instinctively toward a deep pool of water, called by the villagers the Black Pool—so called because of its depth. Near it, and overshadowing it, grew an old gnarled thorn-bush, which, after many winters' frosts and snows, still preserved its vitality. It was a pleasant place in summer. He was found drowned! Every means were used for his restoration, while old Walter was sent off on the brown mare to the doctor's. We heard the dull, heavy sound of her hoofs upon the snow, as she went off at a swift pace down the carriage-drive. In a short time she came back, bringing the doctor.

"My mother was bending over Willie, and nervously swaying herself backward and forward, when he came in; but she arose immediately, and with wide, flashing eyes, cried:

"'O doctor! save my boy! O Willie! Willie darling! Speak to me, my child!'

"I never read David's thrilling lament, 'O Absalom! my son, Absalom!' without thinking of my mother's great agony in Willie's chamber. The doctor was a remarkably skillful man; but it seemed a hopeless case. How my mother's eager eye followed all his movements

"At last, when we were about despairing, Willie gently opened his eyes—those magnificent eyes of his! There was an unspeakable ecstasy on my mother's face, the like of which I have never seen since and never expect to see again. It was coming light when the doctor left us, and Willie was in a refreshing sleep.

"The many-colored rainbow of hope now hung over the vicarage — alas! soon to fade away, leaving us but the cold rain and dark clouds of a great sorrow.

"After an hour or two of sleep, Willie awoke, and told my mother how he heard the shouts and laughter of the children in the drawing-room, and how the music seemed to taunt him; and then how he became afraid, and dared not look where the shadows lay in the library; and how, as he watched the moon rise through the poplars before the window, he was tempted to climb down the ivy-stems; and how he had wandered to the Black Pool, and been tempted to spring across it to get a bunch of crimson berries that hung from a branch on the other side, thinking he would give them to her; and how he had missed his footing and fallen backward into the pond. Then he told her how he arose to the surface—and how he was falling into a sweet and pleasant slumber at the bottom, with thoughts of her passing dream-like through his mind—and how he felt some hand touch him, and an exquisite sensation of pain as if he were dying —and that was all he knew.

"How my mother wept and smiled, clasped him to her bosom, and called him her darling Willie! I need not tell you how my poor father kissed him and asked—ay, he, the stern disciplinarian, asked — pardon of his own child. Willie, fatigued with his long talk, fell asleep again; but it was a troubled, broken slumber. His cheeks grew crimson, and his breath quick and hot, and he trembled as though he were very cold.

"The doctor came again, but this time he shook his head, and said there was no chance for him. My mother and father watched him night and day ; but he grew worse and worse Now he would talk of the wild bees' nests he had found, a few days ago, in a bank in the wood; then he would shout, as if at play; and then, whilst my father covered his face with his hands, and the big tears trickled through his fingers in an agony of grief, he would try to repeat his Latin, and failing to do so correctly, he would begin again, saying in beseeching tones: 'O papa! forgive me! I can not!'

"Willie died one morning, just as the old year was dying amidst frost and snow, repeating his Latin lesson, as my mother held his head with its splendid dark locks on her bosom, and his little hand lay in my father's trembling palm.

